# Dopamine D_4_ Receptor Is a Regulator of Morphine-Induced Plasticity in the Rat Dorsal Striatum

**DOI:** 10.3390/cells11010031

**Published:** 2021-12-23

**Authors:** Alicia Rivera, Diana Suárez-Boomgaard, Cristina Miguelez, Alejandra Valderrama-Carvajal, Jérôme Baufreton, Kirill Shumilov, Anne Taupignon, Belén Gago, M. Ángeles Real

**Affiliations:** 1 Facultad de Ciencias, Instituto de Investigación Biomédica, Universidad de Málaga, 29071 Málaga, Spain; boomgaard83@gmail.com (D.S.-B.); ale_valde@uma.es (A.V.-C.); kirill@wustl.edu (K.S.); mar@uma.es (M.Á.R.); 2 Department of Pharmacology, Faculty of Medicine and Nursing, University of the Basque Country (UPV/EHU), 48940 Leioa, Spain; 3 Institut des Maladies Neurodegeneratives, Université de Bordeaux, UMR 5293, 33000 Bordeaux, France; jerome.baufreton@u-bordeaux.fr (J.B.); anne.taupignon@u-bordeaux2.fr (A.T.); 4 Institut des Maladies Neurodegeneratives, CNRS, UMR 5293, 33000 Bordeaux, France; 5 School of Medicine, Washington University in St. Louis, St. Louis, MO 63110, USA; 6 Facultad de Medicina, Instituto de Investigación Biomédica, Universidad de Málaga, 29071 Málaga, Spain; bgago@uma.es

**Keywords:** dopamine, morphine, addiction, dopamine D_4_ receptor, caudate putamen, plasticity, receptor–receptor interaction

## Abstract

Long-term exposition to morphine elicits structural and synaptic plasticity in reward-related regions of the brain, playing a critical role in addiction. However, morphine-induced neuroadaptations in the dorsal striatum have been poorly studied despite its key function in drug-related habit learning. Here, we show that prolonged treatment with morphine triggered the retraction of the dendritic arbor and the loss of dendritic spines in the dorsal striatal projection neurons (MSNs). In an attempt to extend previous findings, we also explored whether the dopamine D_4_ receptor (D_4_R) could modulate striatal morphine-induced plasticity. The combined treatment of morphine with the D_4_R agonist PD168,077 produced an expansion of the MSNs dendritic arbors and restored dendritic spine density. At the electrophysiological level, PD168,077 in combination with morphine altered the electrical properties of the MSNs and decreased their excitability. Finally, results from the sustantia nigra showed that PD168,077 counteracted morphine-induced upregulation of μ opioid receptors (MOR) in striatonigral projections and downregulation of G protein-gated inward rectifier K^+^ channels (GIRK1 and GIRK2) in dopaminergic cells. The present results highlight the key function of D_4_R modulating morphine-induced plasticity in the dorsal striatum. Thus, D_4_R could represent a valuable pharmacological target for the safety use of morphine in pain management.

## 1. Introduction

Long-lasting use of drugs of abuse elicits persistent molecular and cellular neuroadaptive changes within discrete brain regions—e.g., ventral tegmental area (VTA), ventral and dorsal striatum, prefrontal cortex, amygdala, or hippocampus—which have been identified as the neural substrate for behavioral abnormalities driving addiction, drug craving, and relapse [1]. Such drug-mediated alterations affect several neurotransmitters systems—e.g., dopaminergic, GABAergic or glutamatergic systems—signal transduction pathways, neuronal activity, cellular architecture remodeling and synaptic strength [2,3,4,5,6]. Although there is an extensive amount of data regarding drug-mediated modifications, yet it is a major challenge to understand how this wide variety of neuroadaptations converge and operate together in the different stages of the addiction cycle—binge/intoxication’, ‘withdrawal/negative affect’, and ‘preoccupation/anticipation’ [1].

Most of the studies in drug-induced neuroadaptations have preferentially focused in the traditional areas of the reward system—i.e., mesolimbic dopaminergic pathway arising from the VTA which provides projections into the nucleus accumbens (NAc), the major component of the ventral striatum. However, nowadays major attention is also being given to the caudate putamen (CPu)—dorsal striatum—since this region play a pivotal role in goal-directed behavior, drug-related habit learning and automaticity of drug consumption [7,8,9]. Therefore, it has been hypothesized that the transition from recreational drug use to compulsive drug abuse, and thus the consolidation of drug-related instrumental behaviors, comprises a transition from the ventral to the dorsal areas of the striatum activity [10].

Morphine is one of the most powerful analgesic drugs used to relieve pain though highly addictive, making difficult to design a proper prescription schedule. Indeed, long-term morphine consumption promotes neurobiological adaptations including synaptic and structural plasticity in some brain regions, which ultimately contribute to the development of addiction [5,11]. So far, it has been reported that morphine produces neuronal morphological changes, such as alterations of the dendritic arbor complexity and dendritic spines at nucleus accumbens (NAc) medium spiny neurons (MSNs) and at cortical and hippocampal pyramidal cells [2,5,12,13,14,15]. Chronic morphine treatment also produces neuro-adaptative changes in dopamine neurons of the ventral tegmental area (VTA) and substantia nigra *pars compacta* (SNc), which correlates with altered neuron excitability and dopamine efflux [16,17,18]. Though not as extensively investigated as the NAc, the CPu also undergoes a morphine-induced dysregulation of nigral dopamine inputs and signaling [16,19,20,21]. However, there is no evidence on whether this drug potentially produces morphological plasticity on dorsal striatal MSNs similar to those described in other brain regions as described above.

The identification of novel strategies to suppress the addictive properties of morphine has emerged as a hot topic in the current investigation in opioid addiction [22]. Several studies have highlighted the dopamine D_4_ receptor (D_4_R) as a key modulator in the addicted behavior, since the deficiency in this receptor seems to increase the susceptibility to the development of drugs abuse [23,24]. Of high interest is the evidence that the selective stimulation of the D_4_R decreases morphine-induced hyperlocomotion, reward and withdrawal syndrome, without interfering with the analgesic properties of morphine [16]. These results were strengthened by the demonstration that D_4_R stimulation counteracts morphine-induced activation of the nigrostriatal pathway leading to a restoring of dorsal striatal dopamine tone, and also by the ability of the D_4_R to modulate molecular alterations and long-term μ opioid receptor (MOR) sensitization in the CPu [16,25,26,27]. Despite these evidences, the exact molecular mechanisms by which D_4_R appears to prevent the addictive effects of morphine are not yet fully understood, but an antagonistic D_4_R-MOR interaction in the CPu might occur through the formation of heteroreceptor complexes [16,28,29].

In this perspective, the aim of the present study was to determine how the continuous exposure to morphine would induce structural plasticity and changes in the intrinsic membrane excitability and firing properties of the dorsal striatal MSNs, and whether these neuroadaptations were modulated by D_4_R activation.

## 2. Materials and Methods

### 2.1. Animals

Experiments were conducted in 1–2 month-old male Sprague-Dawley rats (Charles River, Barcelona, Spain). The animals were maintained on a standard light/dark cycle (12/12 h), constant room temperature (20 ± 2 °C) and relative humidity (65 ± 75%) with food and water available ad libitum. Animal care and procedures were conducted in accordance with protocols approved by the Ethical Committee of the University of Málaga (CEUMA 79-2019-A) and the University of Bordeaux (CEEA-50), guidelines from the European Union Council Directive 86/609/EEC, as well as the Spanish Government (R.D. 53/2013).

### 2.2. Drug Administration

Morphine sulfate was obtained from Alcaliber S.A. (Madrid, Spain) subsequent to receiving authorization from Spanish Agency of Medicines and Medical Devices (Spanish Government). PD168,077 maleate was supplied by Tocris Bioscience (Avonmouth, UK). The specificity of PD168,077 as a D_4_R agonist has been extensively proved in prior works using the highly selective antagonist L745,870 [16,25,26,27]. All drugs were dissolved in the vehicle solution consistent in 2% dimethyl sulfoxide (DMSO) and 0.9% NaCl. We have demonstrated in a previous report that this amount of DMSO had no effect on receptors function [30].

Rats received 7 or 14 days of continuous administration of vehicle, morphine (20 mg/kg/d) and/or PD168,077 (1 mg/kg/d) by an osmotic pump (Alzet^®^ osmotic pumps, Cupertino, CA, USA) that was subcutaneously implanted under deep anesthesia (75 mg/kg ketamine and 0.5 mg/kg medetomidine, i.p.) between the shoulder blades. Skin was sutured with sterile non absorbable suture and the surgical site was disinfected with a topical antiseptic (povidone iodine 10% solution). During the surgery and recovery, animals were kept warn using a heating pad. Animals were sacrificed the last day of the continuous administration of drugs.

### 2.3. Electrophysiology

#### 2.3.1. Slice Preparation

Rats (*n* = 19) were sacrificed by decapitation under deep anesthesia (4% isoflurane) after 14 days of continuous drugs treatments. Brains were removed and transferred into an ice-cold artificial cerebrospinal fluid (ASCF), equilibrated with 95% O_2_ and 5% CO_2_, and containing in mM: 250 sucrose, 26 NaHCO_3_, 7 MgCl_2_, 2 KCl, 1.15 NaH_2_PO_4_, 0.5 CaCl_2_, 1 glucose at pH 7.4. Coronal brain sections (350 μm thick) at the rostral CPu level (+1.7 to +1.20 mm from Bregma) were obtained with a microtome (VT1200S; Leica Microsystems, Germany) and left in ACSF containing (in mM): 124 NaCl, 26 NaHCO_3_, 1.3 MgCl_2_, 3.6 KCl, 2.4 CaCl_2_, 1.25 HEPES, 10 glucose, pH 7.4 gassed with 95% O_2_, 5% CO_2_ until further recording.

#### 2.3.2. Whole-Cell Current-Clamp Recordings

Each section was transferred into a recording chamber and was continuously perfused with an oxygenated ACSF at a rate of 3.5 mL/min. MSNs within the dorsolateral part of the CPu were identified by their morphological characteristics under infrared differential interfere contrast (IR-DIC) optics (Zeiss examiner Z.1). 

The patch pipette was filled with a KGluconate-based solution containing (in mM): 140 KGluconate, 3.8 NaCl, 1 MgCl_2_, 10 HEPES, 0.1 Na_4_EGTA, 2 Mg, 1.5 ATP, and 0.4 Na_3_GTP. The day of the experiment the solution was supplemented by biocytin (2.5–5 mg/mL) and by Alexa fluor 488 (2 μM) to allow morphological analysis of the recorded cells. The pH and osmolarity of the pipette solution were 7.3 and 290 mOsm, respectively. The junction potential between the electrode solution and the external media (empirically estimated as 13 mV) was not corrected. Electrode signals were low-pass filtered at 4 kHz and sampled at 20 kHz. Only one neuron was recorded per slice to avoid uncertainty in the reconstruction phase.

The recordings usually begun in the voltage clamp mode. The neurons were maintained at −80 mV, and 5 successive voltage steps of −10 mV were applied. The average current response was analyzed off-line and cell capacity (Cm), membrane resistance (Rm) and access resistance (Ra) were calculated. In some neurons, only current clamp was used, and these three parameters were calculated from the voltage response to a −25pA stimulation. In the current clamp mode, incremental currents from −300 to +500 pA were injected in 25 or 50 pA steps to explore the subthreshold and firing properties of the neurons. Off line analysis was performed using pClamp V9.2 (Molecular Devices, San Jose, CA, USA), Origin V7 (OriginLab, Northampton, MA, USA), Prism5 (GraphPad Software, San Diego, CA, USA) and R (R Foundation for Statistical Computing, Austria). After recording, the slices were rinsed, fixed in paraformaldehyde (2%), and kept frozen at −20 °C until biocytin revelation.

### 2.4. Cell Reconstruction and Morphometric Analysis

After the recordings, sections were fixed with 4% paraformaldehyde, rinsed with 0.1 M phosphate-buffered saline, pH 7.4 (PBS) and incubated with streptavidin-conjugated Alexa Fluor^®^ 488 (Table S1) diluted 1:500 in PBS with 0.1% Triton X-100 and 0.02% sodium azide for 24 h in the dark. After washing with PBS, the sections were mounted on glass slides and coverslipped with an aqueous-based mounting medium. 

Only completely Alexa Fluor^®^ 488 filled neurons were analyzed. Serial optical sections (0.1 μm step size) of each neuron were acquired with a laser-scanning confocal microscope (Leica, TCS NT) using a 63 × 1.3 numerical aperture oil-immersion. Images were acquired at high-resolution, with an image size of 1024 × 1024 pixels, and a voxel size of 0.27 × 0.27 × 1 μm for the *x*, *y*, and *z* axes, respectively. Image stacks were deconvolved to reduce signal blurring and each cell were three-dimensional reconstructed, traced, and analyzed using the image analysis system NeuronStudio (Icahn School of Medicine at Mount Sinai) [31]. For Sholl analysis, the number of intersections were counted between dendritic branches and a series of concentric spheres, starting at 10 μm from the soma and at radial increment of 1 μm. To quantify spine density, a digital zoom (6.7-fold augmentation) was applied and at least 10–15 dendritic segments (10 μm length) per branch order from each neuron were obtained. Each segment was manually inspected and appropriate corrections made using the NeuronStudio interface. Dendritic protrusions (thin, stubby, and mushroom spines) were classified according to their shapes: mushroom with large head and short neck; thin with thin head and long neck; and stubby with large head and no apparent neck.

### 2.5. Immunohistochemistry

Animals (*n* = 24) were transcardially perfused with 0.1 M PBS followed by 4% paraformaldehyde under deep sodium pentobarbital anesthesia (60 mg/kg, i.p.). The brains were rapidly removed, overnight post-fixed in the same fixative, cryoprotected in 30% sucrose in PBS (72 h) and frozen in dry ice. Rostro-caudal series of coronal sections (30 μm thick) were obtained with a freezing microtome (CM 1325, Leica, Weztlar, Germany) and stored in PBS containing 0.02% sodium azide. Free-floating sections were taken from the CPu (+1.0 to −0.30 mm from Bregma) and substantia nigra (−5.30 to −5.80 mm from Bregma) and processed for either single or double immunolabeling procedures. 

Details regarding the antibodies used in the present study are listed in Table 1 (primary antibodies) and Table S1 (secondary antibodies). All primary antibodies were diluted in phosphate-buffered saline containing 0.2% Triton X-100 (PBS-TX) and 0.1% sodium azide.

#### 2.5.1. Single Immunohistochemical Labeling

Free-floating brain sections were pre-treated for 15 min with 3% H_2_O_2_, rinsed in 0.1 M PBS and then incubated in the primary antibody (Table 1) for 24–48 h at room temperature. After being rinsed in PBS, the sections were incubated for 1 h in the appropriate secondary biotinylated antibody (Vector Laboratories, Burlingame, CA, USA) (Table S1) diluted 1:500 in PBS-TX. The sections were washed again with PBS and incubated for 1 h in peroxidase-conjugated streptavidin (Sigma-Aldrich, St. Louis, MO, USA) diluted 1:2000 in PBS-TX. Peroxidase activity was developed with 0.05% 3,3´-diaminobenzidine (DAB, Sigma-Aldrich, St. Louis, MO, USA) in the presence of 0.02% H_2_O_2_ and 0.08% nickel ammonium sulfate. Sections were mounted on gelatin-coated slides, air dried, dehydrated, and coverslipped with DPX mounting medium.

#### 2.5.2. Double Immunohistochemical Labeling

Selected sections were sequentially incubated with the two primary antibodies of interest for 24–48 h at room temperature each. After being rinsed in PBS, the sections were incubated for 1 h at room temperature in a mixture of the appropriate secondary antibodies conjugated with Alexa 488 or Alexa 568 (Thermo Fisher, Waltham, MD, USA) (Table S1) yielding a green or red fluorescent signal, respectively. At the end of the staining, the sections were mounted on glass slides, coversliped with an aqueous-based mounting medium and observed with a Leica SP8 laser confocal microscopy (Leica, Wetzlar, Germany).

#### 2.5.3. Microscopic Analysis and Semi-Quantification

Semi-quantitative analyses of optical density (OD) of immunoreactivity (IR) were performed, as described elsewhere [30], using the software ImageJ 1.48v (National Institutes of Health, NIH). The measures were performed form gray-scale photomicrographs obtained with a digital camera (DS-Fi1, Nikon, Tokyo, Japan) coupled to an optical microscope (Eclipse E400, Nikon, Tokyo, Japan) (40× objective). All OD values where corrected with the OD from an immunonegative area and data were expressed as mean percentage OD of control.

### 2.6. Western Blot

Animals (*n* = 16) were sacrificed by decapitation and the brains immediately removed. The substantia nigra was dissected and homogenized in a lysis buffer containing 2 mM orthovanadate and a mixture of protease inhibitors (Roche Diagnostics GmbH, Switzerland). After incubating on ice for 30 min, samples were centrifuged at 8000× *g* for 10 min at 4 °C. The supernatant was used for western blotting. Equal amounts of protein (50 μg) was loaded and resolved in 12 % SDS-polyacrylamide gel electrophoresis and then transferred onto PVDF membranes (Hybond-P, GE Healthcare, UK). Membranes were incubated overnight at 4 °C with a specific primary antibody (Table 1), followed by HRP-conjugated secondary antibody. Bands of the membranes were visualized using chemiluminescence detection (ECL, GE Healthcare, UK). Comparisons between the experimental groups were performed by determining bands immunoreactivity by densitometry with the image analyzing system ImageJ1.48v (NIH).

### 2.7. Statistical Analysis

Data are expressed as mean ± SEM. Statistical differences (*P* < 0.05) were assessed by one-way analysis of variance (ANOVA) followed by Bonferroni t test or Kruskal–Wallis analysis followed by Dunn’s test for nonparametric data. Statistical analysis and graphs were generated using Prism 5 (GraphPad Software).

## 3. Results

### 3.1. D_4_R Activation Results in MSNs Dendritic Arbors Stretching and Prevention of Morphine-Induced MSNs Dendritic Contraction

**Figure 1:**
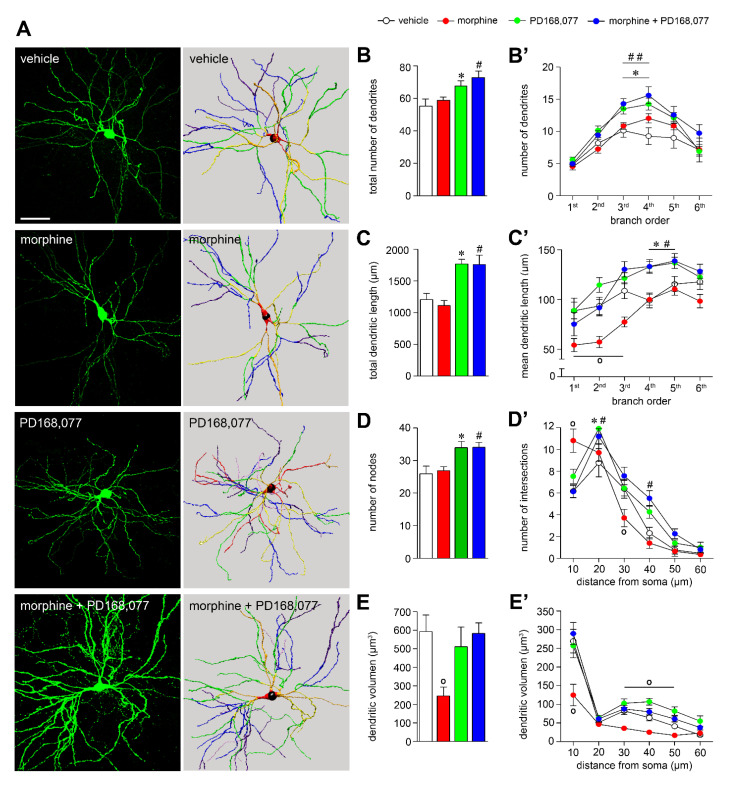
Changes in striatal MSNs dendritic arbor complexity after continuous administration of morphine and/or PD168,077. (**A**) Representative striatal MSNs confocal images (left panels) and reconstructions (right panels) demonstrating the effect of continuous treatment (14 days) with vehicle, morphine (20 mg/kg/d), PD168,077 (1 mg/kg/d) and morphine + PD168,077 (20 mg/kg/d and 1 mg/kg/d). Dendritic branch order is coded by: red, 1st; orange, 2nd; yellow, 3rd; green, 4th; blue, 5th; purple, 6th; pink, 7th. (**B**) Total number and (**B’**) number of dendrites as a function of dendritic branch order. (**C**) Total dendritic length (µm) and (**C’**) dendritic length as a function of dendritic branch order. (**D**) Number of nodes and (**D’**) number of Sholl intersections along the dendritic arbor. (**E**) Total dendritic volume (μm^3^) and (**E’**) Sholl analysis of dendritic volume along the dendritic arbor. Data displayed as mean ± SEM, *n* = 23 neurons on average per treatment. ◦ *p* < 0.05 vehicle vs. morphine; * *p* < 0.05 vehicle vs. PD168,077; # *p* < 0.05, ## *p* < 0.01 vehicle vs. morphine + PD168,077; one-way ANOVA followed by Tukey test (B,B’) or Kruskal-Wallis test followed by Dunn’s method (C,C’,D,D’,E,E’). Scale bar is 50 μm.

Morphine-induced structural plasticity has been previously demonstrated in several brain regions—e.g., NAc, prefrontal cortex or hippocampus [5,15]. To address whether morphine could also produce structural plasticity on striatal projection neurons of the CPu, we first analyzed MSNs dendritic arborization after 14 days of continuous drug treatment (Figure 1A). We found that morphine apparently did not alter the total number of dendrites (Figure 1B), neither the total dendrite length (Figure 1C) nor the number of nodes (Figure 1D). However, there was a pronounced and significant reduction in the dendritic volume (by 60%) (Figure 1E). To further explore the impact of morphine on the dendritic arbor complexity, a morphological analysis was performed considering both the dendritic branch order and the distance from the soma. Morphine treatment produced both a remarkable shrinkage of proximal dendrites as a consequence of its shortening (>60% of reduction in the 1st, 2nd, and 3rd branch order) and volume diminishment (by 50% in the dendrites directly emanating from the soma) (Figure 1C’–E’).

Given the ability of D_4_R activation to prevent morphine-induced impairment of nigral dopaminergic signaling [16] and some of the molecular maladaptive changes related to drug addiction [25,26,27,32], we next studied whether the D_4_R agonist PD168,077 could affect morphine-induced MSNs dendritic arbors remodeling. The continuous administration (14 days) of PD168,077 alone or in combination with morphine yield a prominent expansion of the MSNs dendritic arbors (Figure 1A), which was demonstrated by an overall increase in the number of dendrites (PD168,077: by 23%; morphine + PD168,077: by 33%) (Figure 1B), total dendritic length (PD168,077: by 47%; morphine + PD168,077: by 46%) (Figure 1C), and number of nodes (PD168,077: by 41%; morphine + PD168,077: by 37%) (Figure 1D). Interestingly, these effects were significantly different across the dendritic arbor. Thus, PD168,077 and morphine + PD168,077 did not modify the morphological characteristics of proximal dendrites (1st and 2nd branch order) but increased the complexity of distal dendrites (4th and 5th branch order) (Figure 1B’–D’). As it is shown in Figure 1E–E’, dendritic volume was not altered neither by PD168,077 nor morphine + PD168,077 treatments compared to controls animals.

### 3.2. D_4_R Activation Counteracts Dendritic Spine Depletion Induced by Morphine

**Figure 2:**
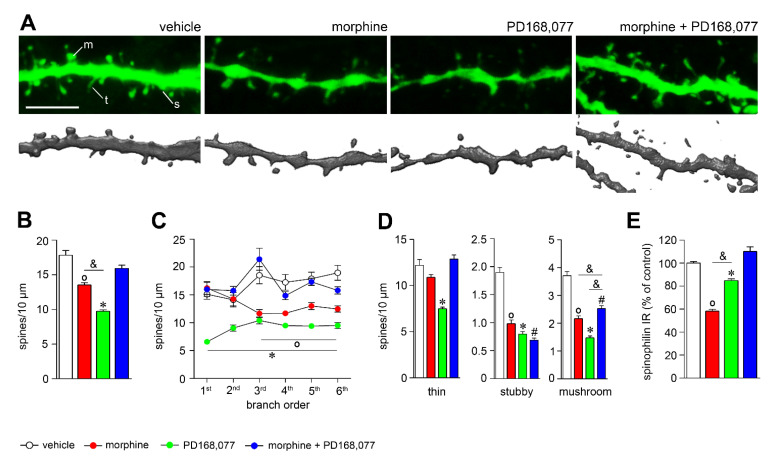
D_4_R activation restores spine density depletion induced by morphine. (**A**) Representative confocal images (upper panels) and reconstructions (lower panels) of dendrites showing the effect of continuous treatment (14 days) with vehicle, morphine (20 mg/kg/d), PD168,077 (1 mg/kg/d) and morphine + PD168,077 (20 mg/kg/d and 1 mg/kg/d) on spine density. (**B**) Overall spine density and (**C**) spine density as a function of dendritic branch order. (**D**) Spine density of the three classified spines (thin, stubby and mushroom) based on shape. (**E**) Histogram shows the semi-quantitative analysis of spinophilin IR in the CPu. Data represents mean ± SEM, *n* = 23 rats per treatment. ◦ *p* < 0.05 vehicle vs. morphine; * *p* < 0.05 vehicle vs. PD168,077; # *p* < 0.05 vehicle vs. morphine+PD168,077; & *p* < 0.05 morphine vs. PD168,077; Kruskal-Wallis test followed by Dunn’s method. Scale bar is 5 μm. Abbreviations: m, mushroom; s, stuby; t, thin.

We next analyzed the effects of the continuous treatment (14 days) with morphine and/or PD168,077 on MSNs spine density (Figure 2A). Spine density was significantly reduced in the striatal MSNs of morphine-treated animals as compared to control rats (by 25%) (Figure 2B). This spine loss was observed in distal dendrites (3rd, 4th, 5th, and 6th branch order) (Figure 2C) and it was associated with the selective reduction in stubby (by 48%) and mushroom (by 42%) spines (Figure 2D). The D_4_R agonist produced an even greater decrease in the overall spine density (by 45%) (Figure 2B), but in this case it occurred throughout the entire dendritic arbor (Figure 2C). The analysis of spine morphology indicated that such PD168,077-induced lower spine density was due to a decrease in the number of thin (by 40%), stubby (by 58%) and mushroom (by 60%) spines (Figure 2D).

When PD168,077 was administered together with morphine, spine density was apparently reestablished (Figure 2A–C), as it was observed for thin-shaped spines (Figure 2D). However, it should be noted that morphine + PD168,077 was unable to restore the loss of stubby spines and only partially did for mushroom spines (Figure 2D).

Since spinophilin is a protein highly enriched in dendritic spines where it plays a key role in modulating spine density and synaptic activity [33], we evaluated its possible regulation by morphine and/or PD168,077. It was showed that the continuous treatment with morphine or PD168,077 alone downregulated striatal levels of spinophilin, which was specifically counteracted by the co-treatment of both drugs (Figure 2E and Figure S1). Thus, the drugs-induced changes in spinophilin protein levels corroborated the modulation of spine density described above.

### 3.3. Changes in Striatal MSN Cell Bodies after Continuous Administration of Morphine and/or PD168,077

**Figure 3:**
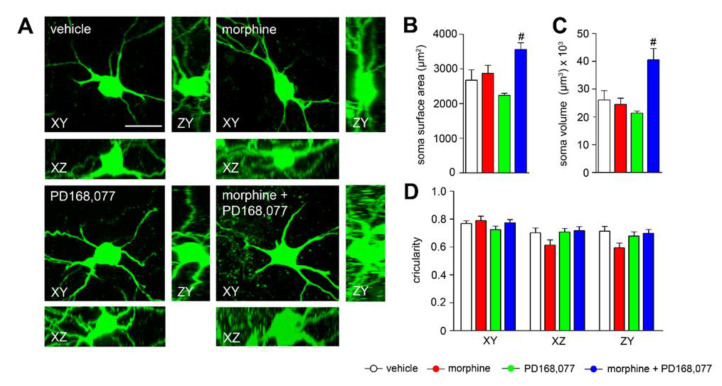
Co-treatment with the D4R agonist and morphine increases the size of the striatal MSNs. (**A**) Representative XY, XZ and YZ confocal images of MSNs soma showing the effect of continuous treatment (14 days) with vehicle, morphine (20 mg/kg/d), PD168,077 (1 mg/kg/d) and morphine + PD168,077 (20 mg/kg/d and 1 mg/kg/d). (**B**,**C**) Histograms represent the effect of drugs treatment on surface area (μm2) and volume (μm3) of the striatal MSNs. (**D**) Graph depicts the circularity (index for soma shape) of MSNs evaluated in the three axis (XY, XZ and XY). Data represents mean ± SEM, *n* = 23 neurons on average per treatment. One-way ANOVA followed by Tukey test, # *p* < 0.05 vehicle vs. morphine + PD168,077. Scale bar is 30 μm.

Given the specific remodeling of the MSNs dendritic arbors and dendritic spines in response to continuous treatment with morphine or/and PD168,077, we next examined whether these changes appear together with morphological alterations in the soma (Figure 3A). We observed that morphine or PD168,077 did not affect the morphology of the striatal MSNs somata, demonstrated by an absence of changes in the size (surface area and volume; Figure 3B,C) and shape (circularity; Figure 3D). However, the combined treatment with both drugs significantly increased the size of the striatal projecting neurons (surface area: by 33%; volume: by 55%) (Figure 3B,C).

### 3.4. D_4_R Activation during Continuous Treatment with Morphine Changes the Passive Properties and Excitability of Striatal MSNs

**Figure 4:**
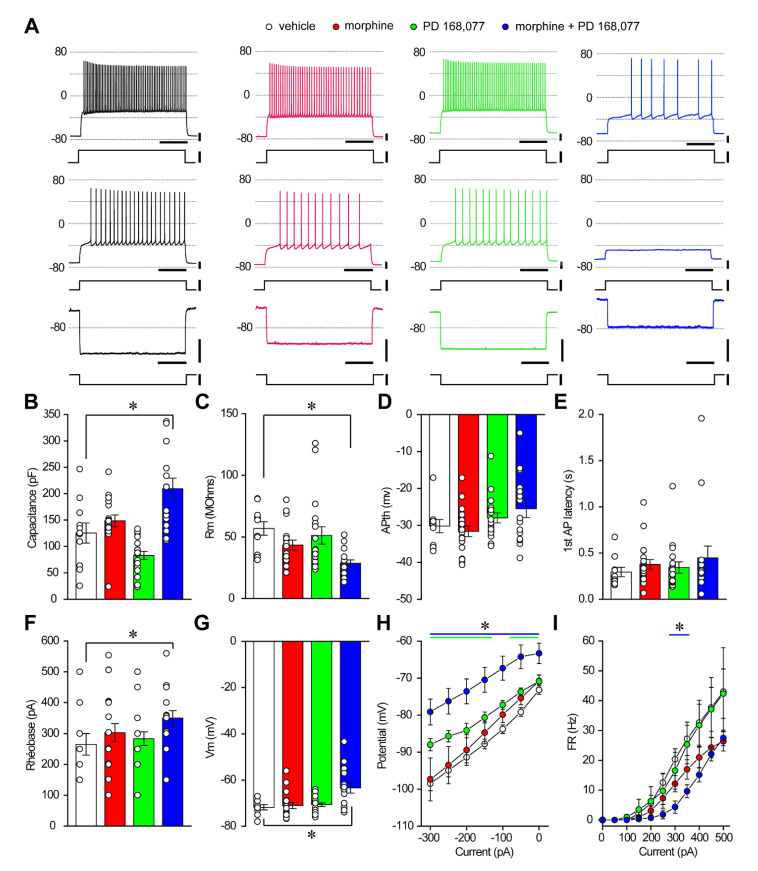
The combined treatment of the D4R agonist PD168,077 with morphine induces changes in passive properties and in excitability of striatal MSNs. (**A**) Representative examples of the voltage responses of identified MSNs of rats treated for 14 days with saline (black), morphine (red; 20 mg/kg/d), PD 168,077 (green; 1 mg/kg/d), and morphine + PD 168,077 (blue, 20 mg/kg/d and 1 mg/kg/d) to two second-long current injection of +300, +200, and −200 pA (top to bottom), respectively. All traces were obtained from neurons that were recorded using a pipette solution complemented with biocytin, and included in the post-recording morphological analysis. (**B**–**G**) Population graphs depicting significant differences in membrane capacitance (**B**), membrane resistance (RM, C), action potential threshold (APth, D), first action potential latency (**E**), rheobase (**F**), and membrane potential (Vm, G) only in the morphine + PD168,077 group. (**H**) Graph showing voltage deflections in response to injection of negative currents (−50 pA steps) in the four experimental groups. (**I**) Graph showing driven activity in response to injection of positive currents (+50 pA steps) in the four experimental groups. Note that current injection evokes significantly milder responses in the morphine + PD showed group. The data only include values obtained from the neurons that were reconstructed and thus included in the post-recording morphological analysis. In **B**–**G**, data were compared using Kruskal-Wallis test followed by a Dunn’s post hoc test. In **H**,**I**, data were compared using mixed model ANOVA followed by Dunnet’s post hoc test * *p* < 0.05.

Since the continuous treatment with morphine and/or PD168,077 regulated dendritic arborization, dendritic spines density and soma size of striatal MSNs, we hypothesized that their electrical activity might be also altered. We used whole-cell patch-clamp recordings to evaluate the intrinsic properties and excitability of striatal MSNs from rats treated for 14 days with vehicle, morphine, PD168,077 or morphine + PD168,077 (Figure 4A). The combined treatment with morphine + PD168,077 yielded significant differences in several passive and active MSNs membrane properties: (i) increased membrane capacitance (Figure 4B); (ii) reduced membrane resistance (Figure 4C); (iii) increased rheobase (Figure 4F); and (iv) depolarized membrane potential (Figure 4G). However, the action potential threshold (Figure 4D) and the first action potential latency (Figure 4E) were not altered by any of the drug treatments.

In addition to the changes in the passive properties, excitability was also reduced in the morphine + PD168,077 group compared with vehicle, morphine or PD168,077. The combined treatment group showed significantly smaller voltage-deflection (Figure 4H) and slower firing frequency (Figure 4I) in response to negative or positive current injection (50 pA steps), respectively.

### 3.5. Regulation of GIRK1 and GIRK2 Expression in the Striatal MSNs and Nigral Dopamine Neurons by Morphine and/or PD168,077 Treatments

Changes in the intrinsic and firing properties of striatal MSNs induced by the co-treatment with morphine + PD168,077 suggest a putative modulation of the heteromeric G protein-gated inward rectifier K^+^ channels (GIRK), since these channels are the predominant channel open at the resting membrane potential [34] and it have been largely involved in the control of neuron excitability [35]. This hypothesis is supported by our previous results demonstrating a deregulation in the gene expression pattern of several GIRK subunits after the acute treatment with morphine and/or PD168,077 [36].

**Figure 5:**
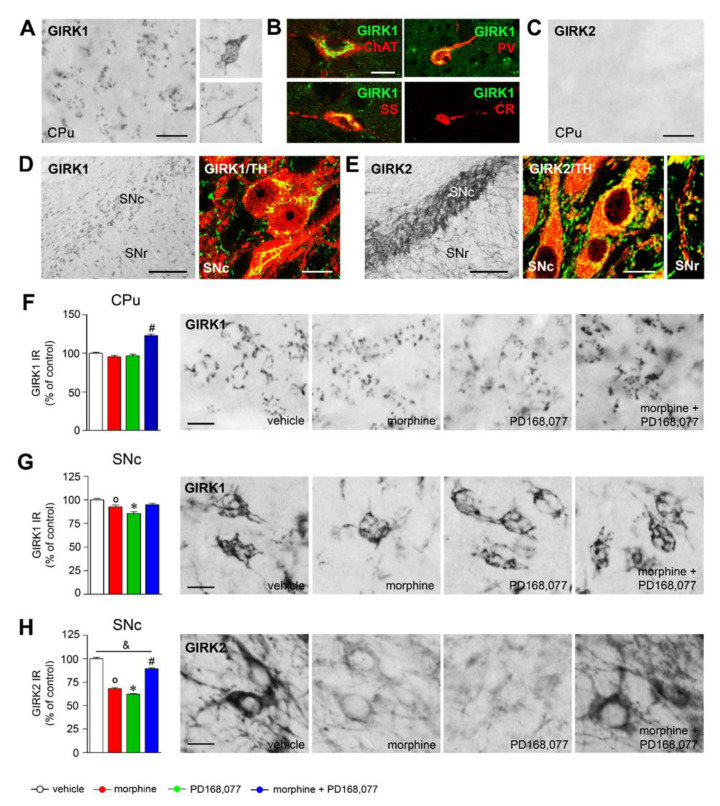
Effects of treatment with morphine and/or PD168,077 on GIRK1 and GIRK2 expression in the CPu and SNc. (**A**) Representative photomicrographs of coronal section of the CPu showing GIRK1 immunoreactivity (IR). The majority of the GIRK1 IR cells are MSNs (left panel in A) displaying a punctate labeling pattern, although few of them are larger cells depicting a reticular-like staining (right panels in A). (**B**) Photomicrographs illustrating by dual labeled immunohistochemistry with anti-GIRK1 (green) and anti-ChAT, anti-PV, anti-SS, and anti-CR (red) the expression of GIRK1 in striatal interneurons. (**C**) Representative photomicrograph showing the absence of GIRK2 expression in the CPu. (**D**,**E**) Photomicrographs illustrating GIRK1 (**D**) and GIRK2 (**E**) expression in the SN and their dual expression with tyrosine hydroxylase (TH). (**F**–**H**) Graphs represent the semi-quantitative analysis of GIRK1 IR in the CPu (**F**) and GIRK1 IR (**G**) and GIRK2 IR (**H**) in the SNc after the continuous (7 days) treatment with morphine (20 mg/kg/d) and/or PD168,077 (1 mg/kg/d). Next to the graphs, representative photomicrographs of GIRK1 and GIRK2 IR in the CPu and SNc are shown. Data represents mean ± SEM, *n* = 6 per treatment per treatment. Kruskal–Wallis test followed by Dunn’s method, ◦ *p* < 0.05 vehicle vs. morphine; * *p* < 0.05 vehicle vs. PD168,077; # *p* < 0.05 vehicle vs. morphine + PD168,077; & *p* < 0.05 morphine vs. morphine + PD168,077. Scale bars are: A,C: 30 μm; B: 10 μm; D,E (left panels): 200 μm; D,E (right panels): 10 μm; F,G,H: 10 μm. Abbreviations: CPu, caudate putamen; SNc, substantia nigra *pars compacta*; SNr, substantia nigra *pars reticulata*.

GIRK1 immunoreactivity (IR) was found in the striatal MSNs displaying a punctate labeling in the cytoplasm (Figure 5A) and in most of the striatal interneurons (PV, SS, and ChAT) showing an intense reticulum-like distribution in the soma and primary dendrites (Figure 5A,B). GIRK2 IR was no detected in the CPu (Figure 5C). Both GIRK1 IR (Figure 5D) and GIRK2 IR (Figure 5E) were present in almost all dopaminergic cells of the SNc identified with an anti-TH antibody. GIRK1 IR showed a reticulum-like distribution in the cell bodies (Figure 5D) whereas GIRK2 was demonstrated in both the perikarya and dendritic branches, even in those that extend down into the substantia nigra *pars reticulata* (SNr) (Figure 5E).

The continuous treatment (7 days) with morphine or PD168,077 did not alter GIRK1 IR in the striatal MSNs. However, the combined administration of both drugs produced a significant increase in GIRK1 IR (by 23%) (Figure 5F). No changes in GIRK1 IR were detected in ChAT interneurons (Figure S2A), which were identified by their morphological characteristics. 

In the SNc, GIRK1 IR and GIRK2 IR were significantly downregulated by morphine (GIRK1: by 10%: GIRK2: by 32%) and PD168,077 (GIRK1: by 15%; GIRK2: by 38%) (Figure 5G,H). However, the co-administration of morphine with PD168,077 completely (for GIRK1; Figure 5G) or partially (for GIRK2; Figure 5H) prevented these effects.

We also observed that the expression of GIRK1 and/or GIRK2 was regulated even after a single dose of morphine and/or PD168,007 in the CPu and SNc, although with a different pattern (Figure S2B).

### 3.6. D_4_R Activation Counteracts Morphine-Induced Upregulation of MOR IR in the Striosome-Dendron Bouquets

**Figure 6:**
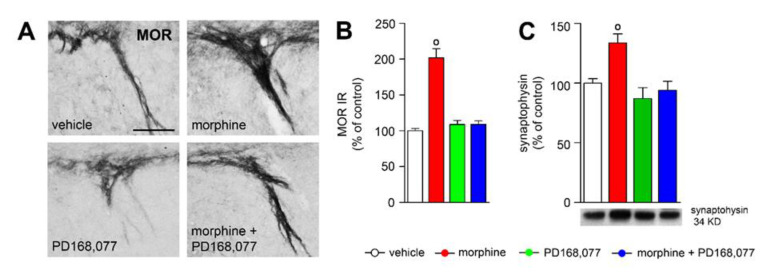
D_4_R agonist treatment counteracts morphine-induced synaptic plasticity in the substantia nigra. (**A**) Representative photomicrographs showing the effect of 7 days of continuous treatment with morphine and/or morphine + PD168,077 on MOR immunoreactivity (IR) in the striosome-dendron bouquets of the sustantia nigra. (**B**) Graph represents the semiquantitative analyses of MOR IR in the striosome-dendron bouquets of the sustantia nigra. (**C**) Histogram shows synaptophysin protein levels in the substantia nigra determined by western blotting analysis. Image are representative of four separate western blots. Data represent mean ± SEM, *n* = 4. ◦ *p* < 0.05 vehicle vs. morphine; Kruskal-Wallis test followed by Dunn’s method (**B**) or one-way ANOVA followed by Tukey test (**C**). Scale bar is 100 μm.

A subset of striatal MSNs originates a direct striatonigral pathway that has been highlighted as crucial modulator of dopamine neuron function in the SNc [37,38,39]. These direct stritatonigral projections target dopaminergic neurons and its ventrally extending dendrites, where they form a specialized integrative unit that has been termed ‘striosome-dendron bouquet’ [40]. High expression of MOR in the striosome-dendron bouquets [40] (and own observation) lead to the speculation that morphine and/or PD168,077 treatments could regulate the levels of this receptor as they do in the CPu [26]. The continuous treatment with morphine (7 days) induced a rise of MOR immunoreactivity (IR) (by 92%) that was completely prevented when morphine was administrated together with PD168,077 (Figure 6A,B).

To obtain more information concerning both synaptic plasticity and transmission in the SN, we used western blot to measure the expression levels of synaptophysin, which is a presynaptic vesicle protein involved in the final steps of exocytosis and synapse formation [41]. Consistent with the previous results described above, it was observed that morphine upregulated synaptophysin (by 30%) in the substantia nigra. The administration of PD168,077 with morphine completely neutralized morphine-induced changes in the expression levels of this protein (Figure 6C).

## 4. Discussion

The purpose of the present study was to determine whether the activation of D_4_R with the specific agonist PD168,077 could modulate morphine-induced neuroplasticity in the CPu. Prolonged exposure to morphine triggered structural plasticity in the dorsal striatal MSNs, which mainly consists of the retraction of the dendritic arbor and the loss of dendritic spines. It is also demonstrated that morphine upregulates MOR in the striatonigral inputs that exert a direct inhibition over nigral dopamine neurons—i.e., striosome-dendron bouquets. On the other hand, the continuous stimulation of D_4_R during morphine treatment also induced structural plasticity in the MSNs, in this case yielding an expansion of the dendritic arbor but restoring dendritic spine density. It was of special interest that the combined treatment of morphine with the D_4_R agonist PD168,077 altered the passive electrical properties and decreases the excitability of the striatal MSNs, along with changes in GIRK channels expression levels and the restoring of MOR expression in the striosome-dendron bouquets.

### 4.1. Morphine-Induced Structural Plasticity in Striatal MSNs

There is strong evidence that morphine elicits structural and functional plasticity mostly in brain regions associated with reward, learning, or incentive motivation, such as the NAc, VTA, PFC, or hippocampus [5,15,42]. These neuroplastic changes persist for long after the discontinuation of morphine treatment. In fact, a recent research has found that morphine-induced alterations in proteins related to synaptic plasticity and cytoskeletal organization persist even six months after cessation of drug administration [43]. However, little was known about whether morphine also produced structural plasticity in the CPu, despite this region is critically involved in drug-related habits formation and consolidation of addiction [1,8,44].

Results of the present study clearly demonstrated that a continuous treatment with morphine triggers a retraction of the dendritic arbor and a significant loss of dendritic spines in the dorsal striatal MSNs. This observation is consistent with previous reports describing a similar pattern of long-term morphine-induced structural plasticity in several regions belonging to the reward circuit [2,5,12,45]. However, more recent studies have highlighted some contradictory data depicting an opposite effect, especially in the NAc, the orbital prefrontal cortex and the dentate gyrus of the hippocampus [13,14,15,46]. These differences could be attributed to several factors, such as the paradigm of drug administration, the animal sacrifice time point, the brain region analyzed, or even the cell type studied.

Interestingly, we observed that the retraction of dendrites specifically occurred by a decrease in the size (length and volume) of the proximal dendrites, while the spines loss was attributable to a selective downregulation of mushroom and stubby spines on distal dendrites. The concurrence of these two forms of structural plasticity could have a major impact on MSNs activity. Firstly, because signals propagation effectiveness throughout dendrites is highly dependent on their morphology [47,48,49,50]. Secondly, because dendritic spines remodeling is tightly linked to synaptic strength, and therefore to the synaptic plasticity phenomena [51]. Thus, the selective loss of mushroom spines after prolonged exposure to morphine could reflects the induction of long-term depression (LTD) processes, as previously demonstrated in excitatory inputs to the dorsal striatum [52,53]. It should be noted that these two structural changes took place in specific subregions of the dendritic arbor, revealing a dichotomy between proximal and distal dendrites. Regarding this, the existence of a topographic organization of the striatal MSNs inputs has been largely suggested although not fully unraveled. Proximal dendrites seem to mainly receive intrinsic shaft connections from striatal interneurons and collateral projections from MSNs, whereas distal dendrites preferentially support extrinsic projections from the cortex, thalamus, or midbrain [54,55]. In this perspective, it is likely that morphine, by promoting morphological changes and therefore affecting synaptic inputs, has a significant impact on the function of the MSNs of the dorsal striatum and ultimately on their output.

Our results show that, although morphine can induce an overall structural plasticity in the MSNs of the CPu, it is unable to modify their passive membrane properties and intrinsic excitability. Interestingly, previous studies have demonstrated that repeated morphine exposure specifically alters the activity of the NAc MSNs which expressed the dopamine D2 receptors (D_2_R) [56,57]. Thus, as in our analysis we did not identify striatal MSNs subpopulations—e.g., direct- and indirect-pathway projection neurons—it remains to be investigated in future works whether morphine specifically mediates changes in one of them. On special interest will also be to reveal the specific action of morphine on the striatonigral projection neurons located in the striosomal compartment since this neural population express MOR and provide direct inputs on nigral dopamine neurons [37,38,39,40,58] and we have observed a morphine-induced increase in MOR expression in the striosome-dendron bouquets.

### 4.2. D_4_R Activation Alter Dendritic Arbor Complexity and Spine Density of the Striatal MSNs

Dopamine action on striatal MSNs through its interaction with dopamine receptors has a major role in both structural and synaptic plasticity occurring in these neurons [59]. In fact, the existence of opposite processes controlling synaptic plasticity induced by dopamine in form of LTP or LTD are postulated to be directed by D_1_R or D_2_R in the direct- and indirect-pathway MSNs, respectively [60,61,62]. These data support the classical model for the independence of the two main striatal pathways outputs [63]. However, this model is currently under review since recent data are quite conflictive. As an example, a recent report has described that D_1_R is essential for the maintenance of spine plasticity in the direct-pathway MSNs but it also affects indirect-pathway neurons [64]. This could be explained on the base of intrinsic connectivity between direct- and indirect-pathways MSNs or the cross-talk of these neurons via interneurons [62]. In addition, several works confirm that a certain proportion of MSNs co-express D_1_R and D_2_R [65,66].

The role of other receptor subtypes different to D_1_R and D_2_R has been largely undervalued in the context of the CPu despite their expression in both direct- and indirect-pathways MSNs. Thus, the existence of D_4_R and D_5_R in the CPu [28,67] opens up the possibility to discover new dopamine signaling mechanisms underlying striatal plasticity. According to our results, we suggest a new role for the D_4_R in mediating the regulation of MSNs morphology by dopamine. Here, we show that prolonged D_4_R stimulation by itself expands the MSNs dendritic arbors, as the activation of these receptors increase both the number of distal dendritic branches and also of their length. This dendritic reshaping could be related to the ability of D_4_R to modulate the expression of the neurotrophic factor BDNF [36] and the transcription factors CREB and ΔFosB [25], which, in turn, regulates the transcription of numerous genes encoding for cytoskeleton regulatory proteins [5]. Paradoxically, D_4_R stimulation also induces a downregulation of all types of dendritic spines (i.e., thin, stubby and mushroom), which occurs across the entire dendritic arbor. However, despite the morphological alterations, the passive membrane properties and excitability remain unchanged. This effect differs from that previously described for D_2_R, since overexpression of this receptor increases the excitability of the MSNs and decreases the complexity and length of their dendritic arbors [68].

### 4.3. D_4_R Activation Modulates Morphine-Induced Plasticity

D_4_R is at present under the focus of interest because of its possible role in cocaine, amphetamine, nicotine, and alcohol addiction [23,24,69,70,71]. It also exists strong evidence that stimulation of the D_4_R with the selective agonist PD168,077 disrupts morphine addiction without affecting its analgesic properties [16]. This effect seems to be the result of an antagonistic receptor–receptor interaction involving a hypothetical D_4_R-MOR heterodimer [16,26], which could exist in several regions were both receptors are co-expressed—striosomal compartment of the CPu and the SNr [28,72].

The current morphological results demonstrated that D_4_R stimulation reversed morphine-induced alterations in thin and mushroom spines, but not in stubby spines. The combined D_4_R agonist and morphine treatment also leads to an expansion of the dendritic arbor that is similar to that observed after the exclusive treatment with the D_4_R agonist. In agreement with these results, transcriptome analysis following acute administration of morphine and PD168,077 provided indications for the modulation of several genes related with dendritic spine dynamic and synaptic formation—*Homer1*, *Pfn2* and *Slitrk1* [73,74,75] that could be also regulated after their chronic administration. Taken together, these results suggest that a cross-talk between D_4_R and MOR could occur to modulate specifically structural plasticity of dendritic spines.

A parallel decrease in MSNs excitability was also observed upon morphine and PD168,077 co-treatments, which was accompanied by related membrane property changes. According with the increase in membrane capacitance, our results showed an enlargement of the MSNs cell surface. On the other hand, changes in the intrinsic excitability could be related with an alteration in the inwardly rectifying K^+^ currents through a modified function of GIRK channels [57] upon MOR and/or D_4_R activation [76,77]. As a proof of concept, we checked changes in the protein expression levels of two of the GIRK channels subunits—GIRK1 and GIRK2—which were previously highlighted by its presumed role in the D_4_R modulation over morphine effects [36]. However, only a significant upregulation of GIRK1 was observed in the CPu after morphine and PD168,077 treatments as no expression change of GIRK2 was found. It is well established that GIRK1 requires the interaction with other GIRK subunits to reach the cell surface and therefore to form functional channels [35,78,79]. Thus, further studies will be needed to clearly characterize the subunits that form the GIRK channel heterotetramers that operates in the dorsal striatal MSNs and its function regarding the regulation of morphine effects.

In addition to the modulatory effect of D_4_R over morphine-induced plasticity in the CPu, we here also observe two indications of neuroadaptative changes in both the nigral dopamine cells and the striatal projections which regulate its function. Firstly, the co-administration of morphine and the D_4_R agonist PD168,077 restores the expression levels of MOR in the GABAergic terminals coming up from the striosomal MSNs which form a network surrounding dopamine nerve cells and its ventrally extending dendrites—‘striosome-dendron bouquet’ [40]. Regarding this, our own previous observation also demonstrated a D_4_R modulation of MOR in the striosomal compartment [26]. Secondly, D_4_R activation counteracts morphine-induced downregulation of GIRK1 and GIRK2 subunits in nigral dopamine cells, which could be associated, as occurs in the hypothalamus, to a reduction in the GIRK channel function [77]. Our hypothesis, which clearly requires further evaluations, is that D_4_R prevents morphine-induced MOR sensitization in the striatal MSNs, leading to a restoring of GABA signaling [27] from the striatum to the SNc which ultimately could reestablish nigral dopamine cell excitability and a normal dopamine output toward the CPu [16,42].

In conclusion, in combination with our previous works that evidence that the stimulation of the D_4_R prevents morphine-induced reward but not analgesia, we have demonstrated that D_4_R appears as a key regulator of morphine-induced plasticity in the CPu. Therefore, it is plausible that D_4_R may disrupts long-term effects of morphine that drive ventral-to-dorsal striatal shifts in addiction consolidation. Thus, pharmacological strategies involving D_4_R activation might take advantages as a potential therapeutic strategy for the safety prescription of morphine for pain relief.

## Figures and Tables

**Table 1:** Primary antibodies used for immunohistochemistry and western blot.

Antibody	Type	Specie	Source / Reference	Dilution
Calretinin (CR)	Poly-	G	Swant (CG1)	1:10,000 (IF)
Choline acetyltransferase (ChAT)	Poly-	G	Millipore (AB144P)	1:750 (IF)
GIRK1 (Kir3.1)	Poly-	R	Alomone Labs (AB2040113)	1:500 (IMQ, IF)
GIRK2 (Kir3.2)	Poly-	R	Alomone Labs (AB2040115)	1:1000 (IMQ, IF)
μ opioid receptor (MOR)	Poly-	R	Millipore (PC165L)	1:50,000 (IMQ)
Parvalbumin (PV)	Mono-	M	Sigma-Aldrich (P3171)	1:5000 (IF)
Somatostatin (SS)	Poly-	G	Santa Cruz (sc-7819)	1:5000 (IF)
Spinophilin	Poly-	R	Millipore (06-852)	1:200 (IMQ)
Synaptophysin	Mono-	M	Abcam (ab8049)	1:2000 (WB)
Tyrosine hydroxylase (TH)	Mono-	M	InmunoStar (P22941)	1:1000 (IF)

Abbreviations: Mono-, monoclonal; Poly-, polyclonal; G, goat; M, mouse; R, rabbit; IMQ: immunohistochemistry; IF: immunofluorescence; WB: western blot.

## Data Availability

The datasets in this study are available from the corresponding author upon reasonable request.

## Supplementary Materials

The following are available online at https://www.mdpi.com/article/10.3390/cells11010031/s1, Figure S1: Changes in spinophilin IR in the CPu after continuous administration (14 days) of morphine and/or PD168,077. Figure S2: Changes in GIRK1 IR in the striatal ChAT interneurons and changes in GIRK1 and GIRK2 IR in the CPu and SNc after acute administration of morphine and/or PD168,077. Table S1: Secondary antibodies used for immunohistochemistry and western blot.
