# Supplementary material for: Dopamine D4 Receptor Is a Regulator of Morphine-Induced Plasticity in the Rat Dorsal Striatum
Source: Cells. 2021 Dec 23;11(1):31. doi: 10.3390/cells11010031 (PMC8750869; doi:10.3390/cells11010031)
Supplement: Supplementary file 1 [file cells-11-00031-s001.zip › Supplementary Figure S1.pdf]

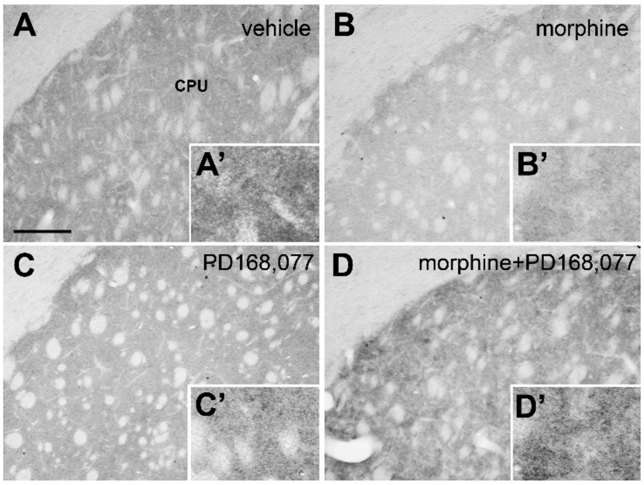

Supplementary Figure S1. Changes in spinophilin IR in the CPu after continuous administration of morphine and/or PD168,077. Representative photomicrographs showing the effect of 14 days of continuous treatment with vehicle (**A**), morphine (20 mg/kg/d) (**B**), PD168,077 (1 mg/kg/d) (**C**) and morphine + PD168,077 (20 mg/kg/d and 1 mg/kg/d) (**D**) on spinophilin immunoreactivity (IR) in the caudate putamen (CPu). (**A′-D′**) High magnification photomicrographs showing punctate spinophilin IR in the CPu. Scale bar is 100 μm for A-D and 40 μm for A′-D′.
