# Supplementary material for: Dopamine D4 Receptor Is a Regulator of Morphine-Induced Plasticity in the Rat Dorsal Striatum
Source: Cells. 2021 Dec 23;11(1):31. doi: 10.3390/cells11010031 (PMC8750869; doi:10.3390/cells11010031)
Supplement: Supplementary file 1 [file cells-11-00031-s001.zip › Supplementary Figure S2.pdf]

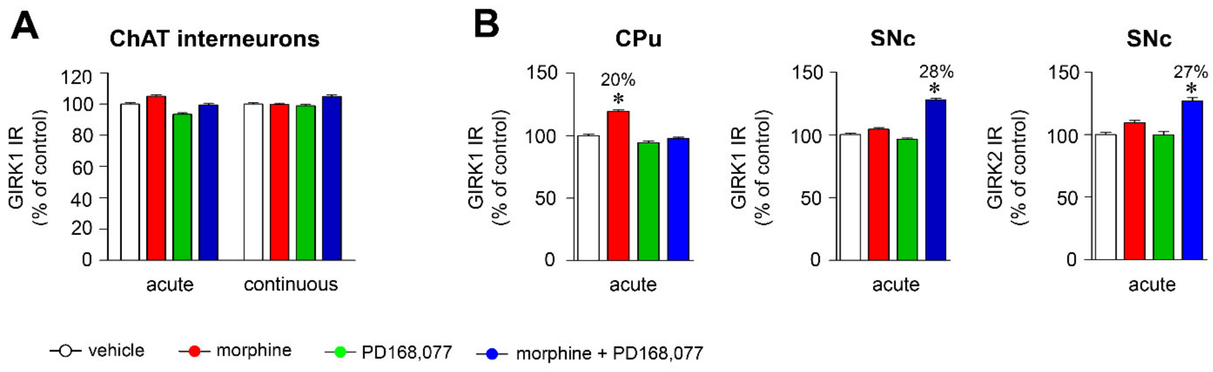

Supplementary Figure S2. Changes in GIRK1 IR in the striatal ChAT interneurons and changes in GIRK1 and GIRK2 IR in the CPu and SNc after acute administration of morphine and/or PD168,077. (**A**) Graph represents the semiquantitative analysis of GIRK1 IR in striatal ChAT interneurons after acute and continuous (7 days) treatment with vehicle, morphine (20 mg/kg/d), PD168,077 (1 mg/kg/d) and morphine + PD168,077 (20 mg/kg/d and 1 mg/kg/d). ChAT interneurons were identified by its morphological characteristics. (**B**) Graphs represent the semiquantitative analysis of GIRK1 IR in the CPu and GIRK1 and GIRK2 in the nigral dopamine cells after acute and continuous (7 days) treatment with vehicle, morphine (10 mg/kg), PD168,077 (1 mg/kg) and morphine + PD168,077 (10 mg/kg and 1 mg/kg). Data represents mean ± SEM, n = 6 rats per treatment. *$P < 0.05$ *vs* vehicle, Kruskal-Wallis test followed by Dunn's method
