# Supplementary material for: Dopamine D4 Receptor Is a Regulator of Morphine-Induced Plasticity in the Rat Dorsal Striatum
Source: Cells. 2021 Dec 23;11(1):31. doi: 10.3390/cells11010031 (PMC8750869; doi:10.3390/cells11010031)
Supplement: Supplementary file 1 [file cells-11-00031-s001.zip › Table S1.pdf]

Table S1. Secondary antibodies used for immunohistochemistry and western blot.

Antibody	Conjugate	Specie	Source	Dilution
**Rabbit IgG**	Biotin	goat	Vector Laboratories	1:500 (IMQ)
**Rabbit IgG**	Alexa 488	donkey	Thermo Fisher	1:1,000 (IF)
**Mouse IgG**	Alexa 568	donkey	Thermo Fisher	1:1,000 (IF)
**Mouse IgG**	HRP	Goat	Vector Laboratories	1:500 (WB)
**Goat IgG**	Alexa 568	horse	Thermo Fisher	1:1,000 (IF)

Abbreviations: IMQ: immunohistochemistry; IF: immunofluorescence; WB: western blot.
